# Hexakis(*N*,*N*-dimethyl­formamide-κ*O*)cobalt(II) bis­(perchlorate)

**DOI:** 10.1107/S160053681000454X

**Published:** 2010-02-10

**Authors:** Frank Eissmann, Tony Böhle, Florian O. R. L. Mertens, Edwin Weber

**Affiliations:** a Institut für Organische Chemie, TU Bergakademie Freiberg, Leipziger Str. 29, D-09596 Freiberg/Sachsen, Germany; b Institut für Physikalische Chemie, TU Bergakademie Freiberg, Leipziger Str. 29, D-09596 Freiberg/Sachsen, Germany

## Abstract

The asymmetric unit of the title complex, [Co(DMF)_6_](ClO_4_)_2_ (DMF = *N*,*N*-dimethyl­formamide, C_3_H_7_NO), consists of two half complex cations with the Co^2+^ metal ions located on centers of inversion and two perchlorate anions. In the crystal packing, each Co^2+^ ion is coordinated by six mol­ecules of DMF in a slightly distorted octa­hedral geometry. The crystal structure is mainly stabilized by coordinative, ionic and C—H⋯O hydrogen-bonding inter­actions.

## Related literature

For the preparation and solution ligand-exchange experiments with [Co(DMF)_6_](ClO_4_)_2_, see: Schneider (1963 ▶); Matwiyoff (1966 ▶); Babiec *et al.* (1966 ▶); Meyer *et al.* (1979 ▶); Męcik & Chudziak (1985 ▶). For other structures containing the [Co(DMF)_6_]^2+^ complex cation, see: Jung *et al.* (1996 ▶); Khutornoi *et al.* (2002 ▶); Guo *et al.* (2004 ▶); Back *et al.* (2007 ▶).
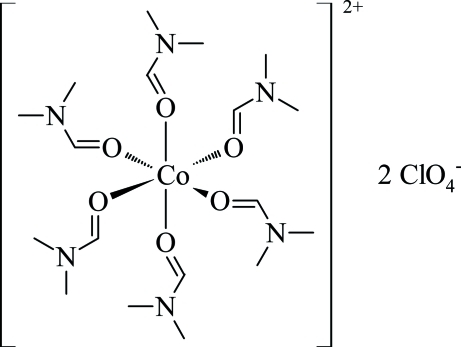


## Experimental

### 

#### Crystal data

[Co(C_3_H_7_NO)_6_](ClO_4_)_2_
*M* *_r_* = 696.41
Monoclinic, 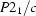

*a* = 14.7573 (3) Å
*b* = 10.7829 (2) Å
*c* = 20.7500 (4) Å
β = 92.265 (1)°
*V* = 3299.29 (11) Å^3^
*Z* = 4
Mo *K*α radiation
μ = 0.75 mm^−1^
*T* = 153 K
0.60 × 0.47 × 0.47 mm

#### Data collection

Bruker Kappa APEXII CCD diffractometer
Absorption correction: multi-scan (*SADABS*; Bruker, 2007 ▶) *T*_min_ = 0.688, *T*_max_ = 0.747
60939 measured reflections
6120 independent reflections
5193 reflections with *I* > 2σ(*I*)
*R*_int_ = 0.024

#### Refinement

*R*[*F* ^2^ > 2σ(*F* ^2^)] = 0.037
*wR*(*F* ^2^) = 0.107
*S* = 1.10
6120 reflections
385 parameters
H-atom parameters constrained
Δρ_max_ = 0.69 e Å^−3^
Δρ_min_ = −0.69 e Å^−3^

### 

Data collection: *APEX2* (Bruker, 2007 ▶); cell refinement: *SAINT* (Bruker, 2007 ▶); data reduction: *SAINT*; program(s) used to solve structure: *SHELXS97* (Sheldrick, 2008 ▶); program(s) used to refine structure: *SHELXL97* (Sheldrick, 2008 ▶); molecular graphics: *SHELXTL* (Sheldrick, 2008 ▶) and *ORTEP-3* (Farrugia, 1997 ▶); software used to prepare material for publication: *SHELXTL* and *PLATON* (Spek, 2009 ▶).

## Supplementary Material

Crystal structure: contains datablocks I, global. DOI: 10.1107/S160053681000454X/su2160sup1.cif

Structure factors: contains datablocks I. DOI: 10.1107/S160053681000454X/su2160Isup2.hkl

Additional supplementary materials:  crystallographic information; 3D view; checkCIF report

## Figures and Tables

**Table 1:** Hydrogen-bond geometry (Å, °)

*D*—H⋯*A*	*D*—H	H⋯*A*	*D*⋯*A*	*D*—H⋯*A*
C1—H1⋯O2^i^	0.95	2.44	3.004 (3)	118
C2—H2*A*⋯O1	0.98	2.39	2.796 (3)	104
C3—H3*C*⋯O14^ii^	0.98	2.37	3.340 (4)	168
C4—H4⋯O10^iii^	0.95	2.42	3.303 (3)	154
C5—H5*A*⋯O2	0.98	2.35	2.762 (3)	105
C5—H5*B*⋯O9^iv^	0.98	2.58	3.549 (4)	171
C8—H8*A*⋯O3	0.98	2.35	2.765 (3)	105
C11—H11*A*⋯O4	0.98	2.36	2.774 (3)	105
C12—H12*C*⋯O9^v^	0.98	2.59	3.397 (4)	140
C13—H13⋯O4	0.95	2.50	3.022 (3)	115
C14—H14*A*⋯O5	0.98	2.39	2.775 (3)	103
C15—H15*A*⋯O11	0.98	2.52	3.482 (4)	168
C15—H15*C*⋯O9	0.98	2.55	3.446 (4)	152
C17—H17*C*⋯O6	0.98	2.32	2.742 (3)	105
C18—H18*A*⋯O2^v^	0.98	2.45	3.371 (3)	156

Symmetry codes: (i) 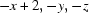; (ii) 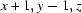; (iii) 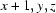; (iv) 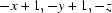; (v) 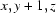.

**Fig. 1.**
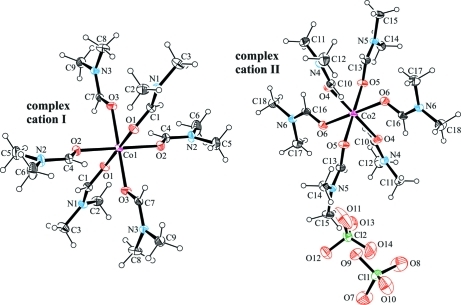
Molecular structure of the title compound with 30 % probability displacement ellipsoids.

**Fig. 2.**
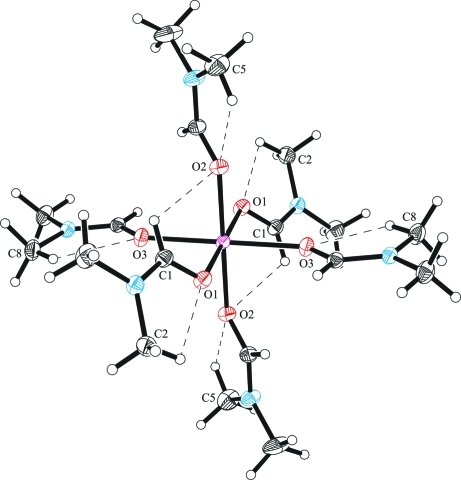
Intramolecular hydrogen bonding interactions (dashed lines) within the complex cations exemplified for complex cation I (Co1).

**Fig. 3.**
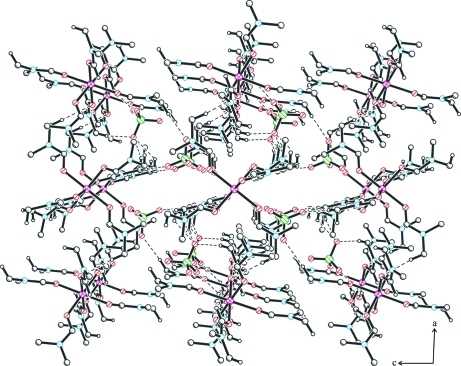
Packing illustration of the title compound viewed down the b axis with intermolecular hydrogen bonding interactions (dashed lines) - see Table 1 for details. Hydrogen atoms not involved in hydrogen bonding interactions have been omitted for clarity.

## supplementary crystallographic information

### Comment

The stability of the title DMF complex concerning ligand exchange in solution was determined under various conditions (Matwiyoff, 1966, Babiec *et al.*, 1966, Meyer *et al.*, 1979 and Męcik & Chudziak, 1985). As described in the literature, the title compound, [Co(DMF)_6_](ClO_4_)_2_, can be prepared as a solid via ligand exchange reaction by adding DMF to an aqueous solution of [Co(H_2_O)_6_](ClO_4_)_2_ (Schneider, 1963). However, an X-ray crystal structure describing the title compound cannot be found in the literature. Taking this into account, suitable crystals of [Co(DMF)_6_](ClO_4_)_2_ were prepared and the crystal structure is reported herein.

The asymmetric unit of the title compound consists of two independent half [Co(DMF)_6_]^2+ ^complex cations and two independent perchlorate anions. Fig. 1 provides an illustration of the asymmetric unit with complete complex cations (produced via the symmetry operators -*x*+2, -*y*, -*z* for complex cation I and -*x*+1, -*y*+2, -*z* for complex cation II). The DMF molecules show a common structure and are coordinated to the Co^2+^ central ion via the carbonyl oxygen atom, respectively. Each Co^2+^ ion is coordinated by six DMF molecules leading to a slightly distorted octahedral complex geometry. The Co—O distances are 2.0687 (14) – 2.1044 (14) Å within complex cation I and 2.0693 (15) – 2.0898 (15) Å within complex cation II. The O—Co—O angles do not deviate significantly from 90° (±3.31° and ±2.69° within complex cations I and II, respectively). The geometrical parameters of the complex cations are in good agreement with the other published X-ray crystal structures containing the [Co(DMF)_6_]^2+^ cation where Co—O distances of 2.05 – 2.16 Å and O—Co—O angles of 90° ± 4° are reported (Jung *et al.*, 1996, Khutornoi *et al.*, 2002, Guo *et al.*, 2004 and Back *et al.*, 2007).

Besides the coordinative interactions between the metal ions and the DMF molecules, the crystal structure of [Co(DMF)_6_](ClO_4_)_2_ is stabilized by several hydrogen bonding interactions (Table 1). The DMF molecules show weak intramolecular (methyl)C—H···O contacts (C2—H2A···O1, C5—H5A···O2, C8—H8A···O3, C11—H11A···O4, C14—H14A···O5 and C17—H17C···O6, Fig. 2). The formyl hydrogens are involved either in weak intra-complex C—H···O interactions with a carbonyl oxygen of another DMF molecule (C1—H1···O2 and C13—H13···O4, Fig. 2) or stronger intermolecular C—H···O contacts to a perchlorate oxygen atom (C4—H4···O10). Furthermore, there are several intermolecular C—H···O contacts between DMF methyl groups and a carbonyl oxygen of a neighboring DMF molecule (C18—H18A···O2) or an oxygen atom of a perchlorate anion (C3—H3C···O14, C5—H5B···O9, C12—H12C···O9, C15—H15A···O11 and C15—H15C···O9). A packing illustration including intermolecular hydrogen bonding interactions is shown in Fig. 3.

### Experimental

Crystals suitable for X-ray analysis were obtained by slow evaporation of a solution of 18.3 mg (0.05 mmol) of [Co(H_2_O)_6_](ClO_4_)_2 _in a mixture consisting of 10 ml of DMF and 10 ml of water.

### Refinement

The H atoms were positioned geometrically and allowed to ride on their respective parent atoms [(formyl)C—H = 0.95 Å and *U*_iso_(H) = 1.2 *U*_eq_(C); (methyl)C—H = 0.98 Å and *U*_iso_(H) = 1.5 *U*_eq_(C)].

### Crystal data

**Table:**

[Co(C_3_H_7_NO)_6_](ClO_4_)_2_	*F*(000) = 1460
*M**_r_* = 696.41	*D*_x_ = 1.402 Mg m^−^^3^
Monoclinic, *P*2_1_/*c*	Mo *K*α radiation, λ = 0.71073 Å
Hall symbol: -P 2ybc	Cell parameters from 9823 reflections
*a* = 14.7573 (3) Å	θ = 2.3–32.2°
*b* = 10.7829 (2) Å	µ = 0.75 mm^−^^1^
*c* = 20.7500 (4) Å	*T* = 153 K
β = 92.265 (1)°	Prismatic, pink
*V* = 3299.29 (11) Å^3^	0.60 × 0.47 × 0.47 mm
*Z* = 4	

### Data collection

**Table:**

Bruker Kappa APEXII CCD diffractometer	6120 independent reflections
Radiation source: fine-focus sealed tube	5193 reflections with *I* > 2σ(*I*)
graphite	*R*_int_ = 0.024
φ and ω scans	θ_max_ = 25.5°, θ_min_ = 2.4°
Absorption correction: multi-scan (*SADABS*; Bruker, 2007)	*h* = −17→17
*T*_min_ = 0.688, *T*_max_ = 0.747	*k* = −13→13
60939 measured reflections	*l* = −25→25

### Refinement

**Table:**

Refinement on *F*^2^	Primary atom site location: structure-invariant direct methods
Least-squares matrix: full	Secondary atom site location: difference Fourier map
*R*[*F*^2^ > 2σ(*F*^2^)] = 0.037	Hydrogen site location: inferred from neighbouring sites
*wR*(*F*^2^) = 0.107	H-atom parameters constrained
*S* = 1.10	*w* = 1/[σ^2^(*F*_o_^2^) + (0.051*P*)^2^ + 2.6993*P*] where *P* = (*F*_o_^2^ + 2*F*_c_^2^)/3
6120 reflections	(Δ/σ)_max_ < 0.001
385 parameters	Δρ_max_ = 0.69 e Å^−^^3^
0 restraints	Δρ_min_ = −0.69 e Å^−^^3^

### Special details

**Table:**

Geometry. All esds (except the esd in the dihedral angle between two l.s. planes) are estimated using the full covariance matrix. The cell esds are taken into account individually in the estimation of esds in distances, angles and torsion angles; correlations between esds in cell parameters are only used when they are defined by crystal symmetry. An approximate (isotropic) treatment of cell esds is used for estimating esds involving l.s. planes.
Refinement. Refinement of *F*^2^ against ALL reflections. The weighted *R*-factor wR and goodness of fit *S* are based on *F*^2^, conventional *R*-factors *R* are based on *F*, with *F* set to zero for negative *F*^2^. The threshold expression of *F*^2^ > σ(*F*^2^) is used only for calculating *R*-factors(gt) etc. and is not relevant to the choice of reflections for refinement. *R*-factors based on *F*^2^ are statistically about twice as large as those based on *F*, and *R*- factors based on ALL data will be even larger.

### Fractional atomic coordinates and isotropic or equivalent isotropic displacement parameters (Å^2^)

**Table:**

	*x*	*y*	*z*	*U*_iso_*/*U*_eq_	
Co1	1.0000	0.0000	0.0000	0.02317 (11)	
N1	1.06651 (12)	−0.03778 (17)	0.19469 (8)	0.0310 (4)	
O1	1.04631 (10)	0.03988 (14)	0.09368 (7)	0.0308 (3)	
C1	1.05875 (14)	−0.0483 (2)	0.13179 (10)	0.0292 (4)	
H1	1.0629	−0.1291	0.1139	0.035*	
C2	1.05863 (18)	0.0810 (2)	0.22680 (11)	0.0412 (6)	
H2A	1.0523	0.1470	0.1944	0.062*	
H2B	1.1131	0.0959	0.2543	0.062*	
H2C	1.0052	0.0804	0.2534	0.062*	
C3	1.0800 (2)	−0.1468 (2)	0.23584 (12)	0.0476 (6)	
H3A	1.0842	−0.2210	0.2089	0.071*	
H3B	1.0287	−0.1552	0.2641	0.071*	
H3C	1.1362	−0.1372	0.2622	0.071*	
N2	0.86908 (13)	0.34263 (17)	0.02245 (10)	0.0375 (4)	
O2	0.90923 (10)	0.14613 (13)	0.00095 (7)	0.0308 (3)	
C4	0.92738 (14)	0.25119 (19)	0.02278 (10)	0.0303 (5)	
H4	0.9867	0.2657	0.0406	0.036*	
C5	0.77732 (17)	0.3280 (3)	−0.00472 (14)	0.0496 (7)	
H5A	0.7704	0.2448	−0.0233	0.074*	
H5B	0.7659	0.3902	−0.0385	0.074*	
H5C	0.7338	0.3390	0.0293	0.074*	
C6	0.8932 (2)	0.4643 (3)	0.04839 (18)	0.0613 (8)	
H6A	0.9566	0.4635	0.0644	0.092*	
H6B	0.8539	0.4843	0.0839	0.092*	
H6C	0.8854	0.5268	0.0144	0.092*	
N3	0.78143 (12)	−0.23773 (16)	0.05297 (8)	0.0290 (4)	
O3	0.90540 (10)	−0.11888 (14)	0.04150 (7)	0.0322 (3)	
C7	0.83944 (14)	−0.17657 (18)	0.01816 (10)	0.0267 (4)	
H7	0.8301	−0.1768	−0.0274	0.032*	
C8	0.79149 (19)	−0.2412 (2)	0.12291 (11)	0.0443 (6)	
H8A	0.8455	−0.1941	0.1370	0.066*	
H8B	0.7980	−0.3274	0.1373	0.066*	
H8C	0.7378	−0.2044	0.1416	0.066*	
C9	0.70395 (15)	−0.3027 (2)	0.02287 (13)	0.0411 (6)	
H9A	0.6478	−0.2597	0.0332	0.062*	
H9B	0.7022	−0.3879	0.0392	0.062*	
H9C	0.7098	−0.3041	−0.0240	0.062*	
Co2	0.5000	1.0000	0.0000	0.03060 (12)	
N4	0.36520 (13)	1.16530 (19)	0.15122 (9)	0.0373 (4)	
O4	0.39957 (10)	1.06505 (15)	0.05968 (7)	0.0356 (4)	
C10	0.41810 (15)	1.1412 (2)	0.10339 (11)	0.0337 (5)	
H10	0.4740	1.1846	0.1020	0.040*	
C11	0.27908 (17)	1.1019 (3)	0.15718 (13)	0.0476 (6)	
H11A	0.2694	1.0445	0.1209	0.071*	
H11B	0.2299	1.1630	0.1568	0.071*	
H11C	0.2798	1.0555	0.1978	0.071*	
C12	0.3916 (2)	1.2536 (3)	0.20153 (13)	0.0536 (7)	
H12A	0.4500	1.2912	0.1917	0.080*	
H12B	0.3973	1.2107	0.2431	0.080*	
H12C	0.3454	1.3186	0.2037	0.080*	
N5	0.42801 (12)	0.68000 (17)	0.09815 (8)	0.0298 (4)	
O5	0.46338 (11)	0.82027 (15)	0.02231 (7)	0.0368 (4)	
C13	0.44254 (15)	0.7930 (2)	0.07824 (10)	0.0317 (5)	
H13	0.4369	0.8587	0.1083	0.038*	
C14	0.43813 (17)	0.5739 (2)	0.05585 (11)	0.0370 (5)	
H14A	0.4380	0.6022	0.0110	0.056*	
H14B	0.3876	0.5163	0.0613	0.056*	
H14C	0.4955	0.5318	0.0668	0.056*	
C15	0.40489 (19)	0.6547 (2)	0.16453 (11)	0.0434 (6)	
H15A	0.4022	0.7328	0.1885	0.065*	
H15B	0.4512	0.6007	0.1849	0.065*	
H15C	0.3457	0.6134	0.1649	0.065*	
N6	0.71229 (12)	1.06214 (18)	0.13840 (8)	0.0325 (4)	
O6	0.58778 (11)	1.00263 (15)	0.08052 (8)	0.0382 (4)	
C16	0.66818 (16)	1.0371 (2)	0.08409 (10)	0.0314 (5)	
H16	0.6993	1.0458	0.0451	0.038*	
C17	0.66574 (19)	1.0597 (3)	0.19895 (11)	0.0543 (7)	
H17A	0.6593	1.1445	0.2151	0.081*	
H17B	0.7010	1.0103	0.2307	0.081*	
H17C	0.6055	1.0226	0.1918	0.081*	
C18	0.80593 (17)	1.1038 (3)	0.14129 (13)	0.0461 (6)	
H18A	0.8328	1.0897	0.0995	0.069*	
H18B	0.8401	1.0573	0.1748	0.069*	
H18C	0.8081	1.1925	0.1516	0.069*	
Cl1	0.14678 (4)	0.44241 (6)	0.15177 (3)	0.04197 (16)	
O7	0.15199 (19)	0.4085 (3)	0.21818 (11)	0.0908 (9)	
O8	0.1056 (2)	0.5590 (2)	0.14163 (16)	0.0996 (10)	
O9	0.23572 (16)	0.4430 (3)	0.12759 (14)	0.0885 (8)	
O10	0.09531 (19)	0.3507 (2)	0.11927 (14)	0.0928 (9)	
Cl2	0.35649 (4)	0.93255 (6)	0.32837 (3)	0.04708 (17)	
O11	0.3907 (3)	0.9056 (3)	0.26930 (13)	0.156 (2)	
O12	0.39639 (17)	0.8536 (2)	0.37650 (11)	0.0723 (6)	
O13	0.37860 (18)	1.0582 (2)	0.34240 (13)	0.0775 (7)	
O14	0.2614 (2)	0.9285 (4)	0.3290 (2)	0.1486 (17)	

### Atomic displacement parameters (Å^2^)

**Table:**

	*U*^11^	*U*^22^	*U*^33^	*U*^12^	*U*^13^	*U*^23^
Co1	0.0272 (2)	0.0197 (2)	0.0226 (2)	−0.00095 (14)	−0.00004 (15)	0.00067 (14)
N1	0.0349 (10)	0.0314 (9)	0.0264 (9)	−0.0034 (8)	−0.0020 (7)	0.0017 (8)
O1	0.0399 (8)	0.0264 (7)	0.0255 (7)	−0.0027 (6)	−0.0048 (6)	0.0003 (6)
C1	0.0323 (11)	0.0261 (10)	0.0289 (11)	−0.0025 (8)	−0.0031 (8)	−0.0008 (9)
C2	0.0532 (14)	0.0402 (13)	0.0307 (12)	−0.0063 (11)	0.0068 (10)	−0.0060 (10)
C3	0.0638 (17)	0.0439 (14)	0.0345 (13)	0.0007 (12)	−0.0049 (12)	0.0111 (11)
N2	0.0332 (10)	0.0276 (10)	0.0513 (12)	0.0070 (8)	−0.0047 (8)	−0.0014 (8)
O2	0.0335 (8)	0.0277 (8)	0.0309 (8)	0.0041 (6)	−0.0012 (6)	−0.0021 (6)
C4	0.0293 (10)	0.0286 (11)	0.0328 (11)	0.0046 (8)	−0.0015 (8)	0.0009 (9)
C5	0.0352 (13)	0.0448 (15)	0.0680 (18)	0.0099 (11)	−0.0094 (12)	0.0059 (13)
C6	0.0535 (17)	0.0330 (14)	0.097 (2)	0.0120 (12)	−0.0098 (16)	−0.0161 (15)
N3	0.0304 (9)	0.0253 (9)	0.0312 (9)	−0.0021 (7)	0.0017 (7)	0.0021 (7)
O3	0.0357 (8)	0.0302 (8)	0.0307 (8)	−0.0081 (6)	0.0013 (6)	0.0035 (6)
C7	0.0304 (10)	0.0234 (10)	0.0264 (10)	0.0029 (8)	0.0004 (8)	0.0023 (8)
C8	0.0650 (16)	0.0369 (13)	0.0316 (12)	−0.0134 (12)	0.0096 (11)	0.0026 (10)
C9	0.0303 (11)	0.0385 (13)	0.0540 (15)	−0.0068 (10)	−0.0048 (10)	0.0067 (11)
Co2	0.0356 (2)	0.0338 (2)	0.0227 (2)	−0.00034 (17)	0.00509 (17)	0.00289 (16)
N4	0.0373 (10)	0.0446 (11)	0.0301 (10)	0.0081 (9)	0.0041 (8)	0.0006 (8)
O4	0.0371 (8)	0.0389 (9)	0.0314 (8)	0.0012 (7)	0.0080 (6)	0.0006 (7)
C10	0.0342 (11)	0.0343 (12)	0.0328 (12)	0.0047 (9)	0.0046 (9)	0.0055 (10)
C11	0.0375 (13)	0.0657 (17)	0.0404 (13)	0.0057 (12)	0.0120 (10)	0.0057 (12)
C12	0.0589 (17)	0.0621 (18)	0.0396 (14)	0.0146 (14)	0.0001 (12)	−0.0141 (13)
N5	0.0364 (10)	0.0321 (9)	0.0215 (9)	0.0018 (8)	0.0070 (7)	−0.0012 (7)
O5	0.0490 (9)	0.0368 (9)	0.0251 (8)	−0.0045 (7)	0.0083 (7)	0.0018 (7)
C13	0.0369 (11)	0.0334 (12)	0.0252 (11)	−0.0003 (9)	0.0052 (9)	−0.0034 (9)
C14	0.0470 (13)	0.0337 (12)	0.0303 (11)	0.0087 (10)	0.0009 (10)	−0.0047 (9)
C15	0.0613 (16)	0.0424 (13)	0.0275 (12)	−0.0021 (12)	0.0151 (11)	0.0034 (10)
N6	0.0365 (10)	0.0399 (10)	0.0216 (9)	0.0062 (8)	0.0053 (7)	−0.0022 (8)
O6	0.0390 (9)	0.0482 (10)	0.0274 (8)	−0.0003 (7)	0.0017 (7)	0.0043 (7)
C16	0.0424 (13)	0.0287 (11)	0.0235 (10)	0.0049 (9)	0.0083 (9)	0.0011 (8)
C17	0.0557 (16)	0.085 (2)	0.0223 (12)	0.0115 (15)	0.0084 (11)	0.0005 (13)
C18	0.0455 (14)	0.0509 (15)	0.0419 (14)	−0.0054 (12)	0.0021 (11)	−0.0067 (12)
Cl1	0.0382 (3)	0.0419 (3)	0.0459 (3)	−0.0083 (2)	0.0034 (2)	−0.0112 (3)
O7	0.0932 (19)	0.137 (3)	0.0429 (12)	−0.0217 (17)	0.0042 (12)	−0.0066 (14)
O8	0.113 (2)	0.0451 (13)	0.144 (3)	0.0159 (14)	0.052 (2)	−0.0069 (15)
O9	0.0543 (13)	0.110 (2)	0.103 (2)	0.0148 (14)	0.0320 (13)	0.0371 (17)
O10	0.105 (2)	0.0667 (15)	0.102 (2)	−0.0213 (14)	−0.0574 (16)	−0.0075 (14)
Cl2	0.0476 (4)	0.0455 (4)	0.0473 (4)	0.0024 (3)	−0.0087 (3)	−0.0001 (3)
O11	0.292 (5)	0.127 (3)	0.0473 (15)	0.129 (3)	−0.002 (2)	−0.0140 (16)
O12	0.0817 (16)	0.0671 (14)	0.0667 (14)	0.0078 (12)	−0.0136 (12)	0.0183 (11)
O13	0.0828 (17)	0.0548 (14)	0.0942 (18)	0.0091 (12)	−0.0049 (14)	−0.0084 (13)
O14	0.0579 (17)	0.150 (3)	0.233 (5)	−0.0344 (19)	−0.047 (2)	0.057 (3)

### Geometric parameters (Å, °)

**Table:**

Co1—O2^i^	2.0687 (14)	Co2—O4	2.0898 (15)
Co1—O2	2.0687 (14)	Co2—O4^ii^	2.0898 (15)
Co1—O1	2.0799 (14)	N4—C10	1.313 (3)
Co1—O1^i^	2.0799 (14)	N4—C11	1.452 (3)
Co1—O3	2.1044 (14)	N4—C12	1.455 (3)
Co1—O3^i^	2.1044 (14)	O4—C10	1.246 (3)
N1—C1	1.311 (3)	C10—H10	0.9500
N1—C2	1.451 (3)	C11—H11A	0.9800
N1—C3	1.462 (3)	C11—H11B	0.9800
O1—C1	1.246 (3)	C11—H11C	0.9800
C1—H1	0.9500	C12—H12A	0.9800
C2—H2A	0.9800	C12—H12B	0.9800
C2—H2B	0.9800	C12—H12C	0.9800
C2—H2C	0.9800	N5—C13	1.307 (3)
C3—H3A	0.9800	N5—C14	1.453 (3)
C3—H3B	0.9800	N5—C15	1.458 (3)
C3—H3C	0.9800	O5—C13	1.247 (3)
N2—C4	1.308 (3)	C13—H13	0.9500
N2—C5	1.455 (3)	C14—H14A	0.9800
N2—C6	1.457 (3)	C14—H14B	0.9800
O2—C4	1.245 (3)	C14—H14C	0.9800
C4—H4	0.9500	C15—H15A	0.9800
C5—H5A	0.9800	C15—H15B	0.9800
C5—H5B	0.9800	C15—H15C	0.9800
C5—H5C	0.9800	N6—C16	1.307 (3)
C6—H6A	0.9800	N6—C18	1.452 (3)
C6—H6B	0.9800	N6—C17	1.456 (3)
C6—H6C	0.9800	O6—C16	1.243 (3)
N3—C7	1.318 (3)	C16—H16	0.9500
N3—C8	1.454 (3)	C17—H17A	0.9800
N3—C9	1.460 (3)	C17—H17B	0.9800
O3—C7	1.238 (2)	C17—H17C	0.9800
C7—H7	0.9500	C18—H18A	0.9800
C8—H8A	0.9800	C18—H18B	0.9800
C8—H8B	0.9800	C18—H18C	0.9800
C8—H8C	0.9800	Cl1—O10	1.403 (2)
C9—H9A	0.9800	Cl1—O8	1.409 (3)
C9—H9B	0.9800	Cl1—O9	1.424 (2)
C9—H9C	0.9800	Cl1—O7	1.425 (2)
Co2—O5^ii^	2.0693 (15)	Cl2—O11	1.375 (3)
Co2—O5	2.0693 (15)	Cl2—O14	1.405 (3)
Co2—O6^ii^	2.0734 (16)	Cl2—O13	1.421 (3)
Co2—O6	2.0734 (16)	Cl2—O12	1.422 (2)
			
O2^i^—Co1—O2	180.00 (10)	O5^ii^—Co2—O4	90.90 (6)
O2^i^—Co1—O1	88.71 (6)	O5—Co2—O4	89.10 (6)
O2—Co1—O1	91.29 (6)	O6^ii^—Co2—O4	92.69 (6)
O2^i^—Co1—O1^i^	91.29 (6)	O6—Co2—O4	87.31 (6)
O2—Co1—O1^i^	88.71 (6)	O5^ii^—Co2—O4^ii^	89.10 (6)
O1—Co1—O1^i^	180.00 (3)	O5—Co2—O4^ii^	90.90 (6)
O2^i^—Co1—O3	88.86 (6)	O6^ii^—Co2—O4^ii^	87.31 (6)
O2—Co1—O3	91.14 (6)	O6—Co2—O4^ii^	92.69 (6)
O1—Co1—O3	86.69 (6)	O4—Co2—O4^ii^	180.00 (7)
O1^i^—Co1—O3	93.31 (6)	C10—N4—C11	121.3 (2)
O2^i^—Co1—O3^i^	91.14 (6)	C10—N4—C12	121.4 (2)
O2—Co1—O3^i^	88.86 (6)	C11—N4—C12	117.2 (2)
O1—Co1—O3^i^	93.31 (6)	C10—O4—Co2	120.82 (14)
O1^i^—Co1—O3^i^	86.69 (6)	O4—C10—N4	124.1 (2)
O3—Co1—O3^i^	180.00 (11)	O4—C10—H10	117.9
C1—N1—C2	121.89 (19)	N4—C10—H10	117.9
C1—N1—C3	121.1 (2)	N4—C11—H11A	109.5
C2—N1—C3	116.96 (19)	N4—C11—H11B	109.5
C1—O1—Co1	118.05 (14)	H11A—C11—H11B	109.5
O1—C1—N1	124.8 (2)	N4—C11—H11C	109.5
O1—C1—H1	117.6	H11A—C11—H11C	109.5
N1—C1—H1	117.6	H11B—C11—H11C	109.5
N1—C2—H2A	109.5	N4—C12—H12A	109.5
N1—C2—H2B	109.5	N4—C12—H12B	109.5
H2A—C2—H2B	109.5	H12A—C12—H12B	109.5
N1—C2—H2C	109.5	N4—C12—H12C	109.5
H2A—C2—H2C	109.5	H12A—C12—H12C	109.5
H2B—C2—H2C	109.5	H12B—C12—H12C	109.5
N1—C3—H3A	109.5	C13—N5—C14	121.39 (18)
N1—C3—H3B	109.5	C13—N5—C15	121.39 (18)
H3A—C3—H3B	109.5	C14—N5—C15	117.17 (18)
N1—C3—H3C	109.5	C13—O5—Co2	120.33 (14)
H3A—C3—H3C	109.5	O5—C13—N5	124.3 (2)
H3B—C3—H3C	109.5	O5—C13—H13	117.9
C4—N2—C5	121.5 (2)	N5—C13—H13	117.9
C4—N2—C6	121.7 (2)	N5—C14—H14A	109.5
C5—N2—C6	116.8 (2)	N5—C14—H14B	109.5
C4—O2—Co1	124.50 (13)	H14A—C14—H14B	109.5
O2—C4—N2	123.5 (2)	N5—C14—H14C	109.5
O2—C4—H4	118.3	H14A—C14—H14C	109.5
N2—C4—H4	118.3	H14B—C14—H14C	109.5
N2—C5—H5A	109.5	N5—C15—H15A	109.5
N2—C5—H5B	109.5	N5—C15—H15B	109.5
H5A—C5—H5B	109.5	H15A—C15—H15B	109.5
N2—C5—H5C	109.5	N5—C15—H15C	109.5
H5A—C5—H5C	109.5	H15A—C15—H15C	109.5
H5B—C5—H5C	109.5	H15B—C15—H15C	109.5
N2—C6—H6A	109.5	C16—N6—C18	122.69 (19)
N2—C6—H6B	109.5	C16—N6—C17	120.3 (2)
H6A—C6—H6B	109.5	C18—N6—C17	116.8 (2)
N2—C6—H6C	109.5	C16—O6—Co2	128.08 (14)
H6A—C6—H6C	109.5	O6—C16—N6	123.7 (2)
H6B—C6—H6C	109.5	O6—C16—H16	118.1
C7—N3—C8	121.13 (19)	N6—C16—H16	118.1
C7—N3—C9	121.35 (19)	N6—C17—H17A	109.5
C8—N3—C9	117.52 (18)	N6—C17—H17B	109.5
C7—O3—Co1	132.08 (14)	H17A—C17—H17B	109.5
O3—C7—N3	123.68 (19)	N6—C17—H17C	109.5
O3—C7—H7	118.2	H17A—C17—H17C	109.5
N3—C7—H7	118.2	H17B—C17—H17C	109.5
N3—C8—H8A	109.5	N6—C18—H18A	109.5
N3—C8—H8B	109.5	N6—C18—H18B	109.5
H8A—C8—H8B	109.5	H18A—C18—H18B	109.5
N3—C8—H8C	109.5	N6—C18—H18C	109.5
H8A—C8—H8C	109.5	H18A—C18—H18C	109.5
H8B—C8—H8C	109.5	H18B—C18—H18C	109.5
N3—C9—H9A	109.5	O10—Cl1—O8	109.58 (19)
N3—C9—H9B	109.5	O10—Cl1—O9	108.8 (2)
H9A—C9—H9B	109.5	O8—Cl1—O9	109.95 (17)
N3—C9—H9C	109.5	O10—Cl1—O7	106.94 (18)
H9A—C9—H9C	109.5	O8—Cl1—O7	112.35 (19)
H9B—C9—H9C	109.5	O9—Cl1—O7	109.08 (17)
O5^ii^—Co2—O5	180.0	O11—Cl2—O14	113.8 (3)
O5^ii^—Co2—O6^ii^	89.59 (6)	O11—Cl2—O13	107.2 (2)
O5—Co2—O6^ii^	90.41 (6)	O14—Cl2—O13	104.4 (2)
O5^ii^—Co2—O6	90.41 (6)	O11—Cl2—O12	110.06 (17)
O5—Co2—O6	89.59 (6)	O14—Cl2—O12	111.2 (2)
O6^ii^—Co2—O6	180.00 (9)	O13—Cl2—O12	110.00 (15)
			
O2^i^—Co1—O1—C1	−43.12 (16)	O5^ii^—Co2—O4—C10	−53.21 (17)
O2—Co1—O1—C1	136.88 (16)	O5—Co2—O4—C10	126.79 (17)
O3—Co1—O1—C1	45.82 (16)	O6^ii^—Co2—O4—C10	−142.84 (16)
O3^i^—Co1—O1—C1	−134.18 (16)	O6—Co2—O4—C10	37.16 (16)
Co1—O1—C1—N1	−162.96 (17)	Co2—O4—C10—N4	−164.16 (17)
C2—N1—C1—O1	1.7 (3)	C11—N4—C10—O4	0.7 (3)
C3—N1—C1—O1	179.3 (2)	C12—N4—C10—O4	178.8 (2)
O1—Co1—O2—C4	43.38 (17)	O6^ii^—Co2—O5—C13	−131.66 (17)
O1^i^—Co1—O2—C4	−136.62 (17)	O6—Co2—O5—C13	48.34 (17)
O3—Co1—O2—C4	130.09 (17)	O4—Co2—O5—C13	−38.97 (17)
O3^i^—Co1—O2—C4	−49.91 (17)	O4^ii^—Co2—O5—C13	141.03 (17)
Co1—O2—C4—N2	179.46 (16)	Co2—O5—C13—N5	−172.41 (17)
C5—N2—C4—O2	−0.7 (4)	C14—N5—C13—O5	2.0 (3)
C6—N2—C4—O2	−179.9 (2)	C15—N5—C13—O5	179.3 (2)
O2^i^—Co1—O3—C7	−99.80 (19)	O5^ii^—Co2—O6—C16	−47.83 (19)
O2—Co1—O3—C7	80.20 (19)	O5—Co2—O6—C16	132.17 (19)
O1—Co1—O3—C7	171.43 (19)	O4—Co2—O6—C16	−138.72 (19)
O1^i^—Co1—O3—C7	−8.57 (19)	O4^ii^—Co2—O6—C16	41.28 (19)
Co1—O3—C7—N3	−172.81 (14)	Co2—O6—C16—N6	163.94 (16)
C8—N3—C7—O3	0.1 (3)	C18—N6—C16—O6	−179.2 (2)
C9—N3—C7—O3	179.1 (2)	C17—N6—C16—O6	−4.8 (4)

Symmetry codes: (i) −*x*+2, −*y*, −*z*; (ii) −*x*+1, −*y*+2, −*z*.

### Hydrogen-bond geometry (Å, °)

**Table:**

*D*—H···*A*	*D*—H	H···*A*	*D*···*A*	*D*—H···*A*
C1—H1···O2^i^	0.95	2.44	3.004 (3)	118
C2—H2A···O1	0.98	2.39	2.796 (3)	104
C3—H3C···O14^iii^	0.98	2.37	3.340 (4)	168
C4—H4···O10^iv^	0.95	2.42	3.303 (3)	154
C5—H5A···O2	0.98	2.35	2.762 (3)	105
C5—H5B···O9^v^	0.98	2.58	3.549 (4)	171
C8—H8A···O3	0.98	2.35	2.765 (3)	105
C11—H11A···O4	0.98	2.36	2.774 (3)	105
C12—H12C···O9^vi^	0.98	2.59	3.397 (4)	140
C13—H13···O4	0.95	2.50	3.022 (3)	115
C14—H14A···O5	0.98	2.39	2.775 (3)	103
C15—H15A···O11	0.98	2.52	3.482 (4)	168
C15—H15C···O9	0.98	2.55	3.446 (4)	152
C17—H17C···O6	0.98	2.32	2.742 (3)	105
C18—H18A···O2^vi^	0.98	2.45	3.371 (3)	156

Symmetry codes: (i) −*x*+2, −*y*, −*z*; (iii) *x*+1, *y*−1, *z*; (iv) *x*+1, *y*, *z*; (v) −*x*+1, −*y*+1, −*z*; (vi) *x*, *y*+1, *z*.
